# Association between maternal cervical Ureaplasma urealyticum colonization and adverse perinatal outcomes: a prospective multicenter cohort study

**DOI:** 10.1186/s12884-026-08839-2

**Published:** 2026-02-26

**Authors:** Cuie Chen, Shuangkui Zhao, Jianmiao Hu, Xianhu Fu, Shujun Chen, Jiajun Zhu, Liping Shi, Danqing Chen, Yanping Xu

**Affiliations:** 1 Department of Neonatology, Yiwu Maternity and Children Hospital, Yiwu, Zhejiang China; 2 Department of NICU, Children’s Hospital, Zhejiang University School of Medicine, No.3333, Binsheng Road, Binjiang District, Hangzhou, Zhejiang 310052 China; 3 Department of Obstetrics, Yiwu Maternity and Children Hospital, Yiwu, Zhejiang China; 4 Department of Obstetrics, Ningbo Women and Children’s Hospital, Ningbo, Zhejiang China; 5 Department of Neonatology, Women’s Hospital School of Medicine Zhejiang University, Hangzhou, Zhejiang China; 6 Department of Obstetrics, Women’s Hospital School of Medicine Zhejiang University, No. 1, Xueshi Road, Shangcheng District, Zhejiang 310000 Hangzhou, China

**Keywords:** Ureaplasma urealyticum, Maternal cervical colonization, Ureaplasma urealyticum load, Perinatal outcomes, Preterm infant

## Abstract

**Background:**

The association between maternal cervical Ureaplasma urealyticum (Uu) colonization and adverse perinatal outcomes remains controversial. This study aimed to investigate the relationship between maternal cervical Uu colonization, Uu load during mid-pregnancy, and perinatal outcomes, with a focus on complications in preterm infants.

**Methods:**

This prospective multicenter cohort study was conducted between July 2021 and January 2024 at four tertiary-level maternity and children’s hospitals in Zhejiang Province, China. Pregnant women with ultrasound-confirmed gestational age of 20^+ 0^ to 24^+ 6^ weeks were enrolled, and cervical Uu colonization was assessed by polymerase chain reaction (PCR). Pregnancy outcomes, including spontaneous preterm birth, pregnancy loss, and neonatal complications, were systematically recorded and analyzed.

**Results:**

Among 452 enrolled participants in this prospective multicenter study, 243 (42.5%) tested positive for Uu colonization. The median Uu DNA load among Uu-positive pregnant women was 1.46 × 10^5^copies/ml. The rate of discontinuation of life-sustaining therapy among preterm infants born at 23–25 weeks of gestation was significantly higher in the Uu-positive group compared to the Uu-negative group (3.7% vs. 0.5%, *P* = 0.045). Women with high Uu load (≥ 10⁵ copies/mL) had significantly increased rates of spontaneous preterm birth before 32 weeks, spontaneous preterm birth before 28 weeks, premature rupture of membranes (PROM), and neonatal death, compared to those with low or no colonization (*P* < 0.05). Multivariate analysis identified Uu load ≥ 10⁵ copies/mL as an independent risk factor for preterm birth before 32 weeks of gestation, with an OR of 5.653 (95%CI: 2.032 ~ 15.727).

**Conclusions:**

Maternal cervical Uu load ≥10⁵ copies/mL may be associated with increased risk of spontaneous preterm birth before 32 weeks.

**Trial registration:**

The research protocol was registered in the Chinese Clinical Trial Registry (No. ChiCTR2100044524) on 10 March 2021.

**Supplementary Information:**

The online version contains supplementary material available at 10.1186/s12884-026-08839-2.

## Background

Preterm birth remains a leading global cause of neonatal and under-5 morbidity and mortality, particularly among infants < 32 weeks of gestation who face elevated risks of infection-related, respiratory, and neurological complications [1, 2]. Intrauterine infection is a well-established contributor to spontaneous preterm delivery [3], highlighting the importance of early pathogen identification and targeted management in high-risk pregnancies.

Ureaplasma urealyticum (Uu) is a small, wall-less prokaryote in the class Mollicutes (genome size ~ 0.75–0.95 Mb) that requires urea for growth and hydrolyzes it via urease to produce ammonia, a process potentially contributing to its pathogenicity. Its lack of a cell wall renders it intrinsically resistant to β-lactam antibiotics [4, 5]. Epidemiological studies report that Uu colonizes the vaginal microbiota of 40–80% of asymptomatic healthy women [6, 7], but under certain immunological conditions, it can act as an opportunistic pathogen inducing local or systemic inflammatory responses [8]. Maternal Uu colonization has been linked to adverse obstetric outcomes, including premature rupture of membranes (PROM), histologic chorioamnionitis, spontaneous preterm delivery (< 37 weeks), and perinatal mortality [9–13]. Ureaplasma species are among the most frequently detected microorganisms in amniotic fluid and placental specimens from patients with idiopathic preterm labor [9, 10, 12–14]. However, some studies, such as Tetsuya et al. [15], found no significant differences in adverse outcomes between Uu culture-positive and culture-negative pregnant women with preterm birth risk factors, reflecting ongoing debate regarding its clinical relevance.

Neonates can acquire Uu infection in utero or during delivery, with vertical transmission rates ranging from 18% to 88% [10, 16]. Uu has been detected in respiratory specimens, serum, and cerebrospinal fluid of preterm infants. Infection in neonates has been associated with pneumonia, sepsis, neonatal respiratory distress syndrome (NRDS), bronchopulmonary dysplasia (BPD), necrotizing enterocolitis (NEC), and central nervous system complications [17–19]. A recent meta-analysis by Xu et al. [20] indicated that maternal Uu colonization increases the risk of prematurity-related neonatal complications, though the quality of evidence remains limited and well-designed prospective studies are needed.

Furthermore, the UU load appears to be clinically relevant. High-density colonization (> 10⁵cfu/mL) has been associated with elevated risks of clinical chorioamnionitis and preterm delivery, with a notable proportion of affected infants being very low birth weight (< 1500 g) [7]. Similarly, Kacerovsky et al. [21] reported that Uu DNA load in cervical fluid was significantly higher in women with PROM before 34 weeks complicated by intra-amniotic infection (median 2.8 × 10⁵ copies/mL) compared to those without infection (median 4.7 × 10³ copies/mL). However, the relationship between varying maternal Uu DNA loads and adverse pregnancy outcomes remains incompletely understood.

Importantly, Uu infections during pregnancy often remain asymptomatic until delivery, making timely detection challenging. Therefore, elucidating the correlation between maternal cervical Uu colonization, UU load, and perinatal outcomes is critical. Cervical sampling provides a convenient method for prenatal Uu screening. Accordingly, we conducted a prospective multicenter cohort study to investigate the relationship between maternal Uu colonization and load in the cervical and their impact on pregnancy and neonatal outcomes.

## Materials and methods

### Study population

This multicenter prospective cohort study was conducted at four tertiary-level A maternity and children’s hospitals located in Hangzhou, Ningbo, and Yiwu in Zhejiang Province, China (approval No. 2021-IRB-045). The research protocol was registered in the Chinese Clinical Trial Registry (No. ChiCTR2100044524) on 10 March 2021. Written informed consent was obtained from all study participants prior to enrollment.

From July 2021 to January 2024, pregnant women who underwent routine prenatal examination at 20^+ 0^ w to 24^+ 6^ w of gestation at four tertiary level A maternity hospitals and children’s hospitals in Zhejiang Province, China, were enrolled. Gestational age determination followed standardized protocols combining last menstrual period documentation with first-trimester ultrasonographic biometry. The exclusion criteria included major medical conditions in pregnant women before 20 weeks of pregnancy, such as pregnancy with congenital heart disease, pregnancy with uremia, fetal birth defects detected by prenatal ultrasound, and conceived triplets. Furthermore, in cases where critically ill newborns were involved, the compassionate withdrawal of life-sustaining therapy was implemented only after multidisciplinary prognosis assessments and with fully informed parental consent, strictly following the approved ethical protocols.

### Study procedures

The obstetricians informed pregnant women about the study individually when they attended routine prenatal examinations. At the enrollment visit, researchers administered a questionnaire that recorded sociodemographic, behavioral, and clinical information in an online research Electronic Data Acquisition (ResMan™ Research Manager) software database. At outpatient clinics, obstetricians collected cervical secretion samples by swabbing the external os of the cervix from participants at 20^+ 0^ to 24^+ 6^ weeks of pregnancy. These samples were subsequently analyzed in the laboratory for species-specific identification and quantification of Uu using a fluorescent probe-based polymerase chain reaction (PCR) method (Roche LightCycler 480, Switzerland; Ureaplasma urealyticum Detection Kit, DaAn Gene Co., Ltd., Guangzhou, China). A quantitative threshold of 1000 copies/mL was applied, with results below this level defined as negative and results at or above it defined as positive. For participants with PCR-positive results, cervical Uu culture and antimicrobial susceptibility testing were subsequently performed. Pregnancy outcomes and neonatal complications were systematically monitored. Postnatal follow-up visits were scheduled from birth through 28 days postpartum or until 36 weeks of corrected gestational age, whichever occurred last. Clinical data pertaining to birth outcomes and perinatal period were collected by research physicians through medical records.

All data were prospectively collected by trained obstetricians and research nurses at each participating center using standardized case report forms (CRFs). The collected items included maternal sociodemographic information, behavioral characteristics, medical and obstetric history, laboratory test results, pregnancy complications, delivery outcomes, and neonatal outcomes. All data were entered and uploaded into the ResMan™ Research Manager electronic data acquisition system, which incorporated range and logic checks to ensure accuracy and consistency. All variables and outcomes (cervical insufficiency [22], gestational diabetes mellitus [23], gestational hypertension [24], PROM [25], chorioamnionitis [26], neonatal sepsis [27], Ventilator Associated Pneumonia (VAP) [28], NRDS [29], BPD [30]) were defined according to standardized clinical criteria.

### Study outcomes

The primary outcome of this study was the association between maternal cervical Uu colonization—including Uu load—and spontaneous preterm birth before 32 weeks of gestation.

Secondary outcomes included the associations between maternal cervical Uu colonization—including Uu load—and spontaneous preterm birth before 28 weeks of gestation, PROM, pregnancy loss (spontaneous abortion or stillbirth), neonatal death before discharge, major neonatal complications such sepsis, NRDS, pneumonia and BPD. Spontaneous abortion was defined as pregnancy loss occurring before 20 weeks of gestation, while stillbirth was defined as fetal death at or after 20 weeks of gestation [31].

### Treatment

In accordance with the routine clinical practice [32], pregnant women who are Uu positive but asymptomatic and without any high-risk factors do not require treatment. However, certain clinical conditions may warrant antimicrobial therapy during pregnancy. Empirical macrolide antibiotics were used, or the antimicrobial agent was selected based on culture and susceptibility results. Antimicrobial treatment was considered in cases where colonization was accompanied by clinical symptoms suggestive of cervical infection (e.g., abnormal vaginal discharge, pelvic discomfort, or elevated inflammatory markers). In particular, treatment was recommended for pregnant women with additional risk factors such as cervical insufficiency, a shortened cervix, or prior to undergoing invasive procedures like cervical cerclage [33]. Based on vaginal secretion culture and antimicrobial susceptibility testing, an appropriate macrolide antibiotic was selected for Uu treatment.

### Sample size calculation

This study employed logistic regression analysis to evaluate the association between Uu colonization in the cervical and adverse pregnancy outcomes. Based on previous literature [34, 35], the incidence rate of adverse pregnancy outcomes is approximately 10%, with an OR of 2.37 for the association between cervical Uu colonization and adverse pregnancy outcomes. With an α level of 0.05 and 80% power, the calculated sample size was 432 participants. Sample size estimation was performed using PASS 2021 software.

### Statistical analysis

Statistical analyses were performed via SPSS 25.0 (SPSS Inc., Chicago, IL, USA). Continuous variables are presented as the means ± SD when normally distributed and as the medians (quartiles) when not normally distributed. Categorical variables are expressed as frequencies and percentages. The continuous variables were analyzed by independent sample t test or Mann‒Whitney U test. The applicability of test are as follows: use the independent samples t-test when variances are homogeneous and use the Mann-Whitney U test when variances are non-homogeneous. The categorical variables were analyzed by the chi-square test, corrected chi-square test, or Fisher’s exact probability method. The applicability of test are as follows: when the sample size is ≥ 40 and the theoretical frequency T ≥ 5, the basic formula of Chi-square test was used; when the sample size is ≥ 40, but the theoretical frequency 1 ≤ T < 5, the corrected Chi-square test was used; when the sample size is < 40, or the theoretical frequency T < 1, Fisher’s exact probability method was used. Multivariate logistic regression analysis was performed to identify independent risk factors for the primary outcome (preterm birth before 32 weeks of gestation). The multicollinearity of variables was assessed by the variance inflation factor (VIF). Variables with *P* < 0.05 in univariate analyses were entered into the multivariate model. Odds ratios (ORs) with 95% confidence intervals (CIs) were calculated. A two-sided *P* < 0.05 was considered statistically significant.

## Results

### Maternal Characteristics and Perinatal Outcomes in Relation to cervical Ureaplasma urealyticum Colonization

**Fig. 1:**
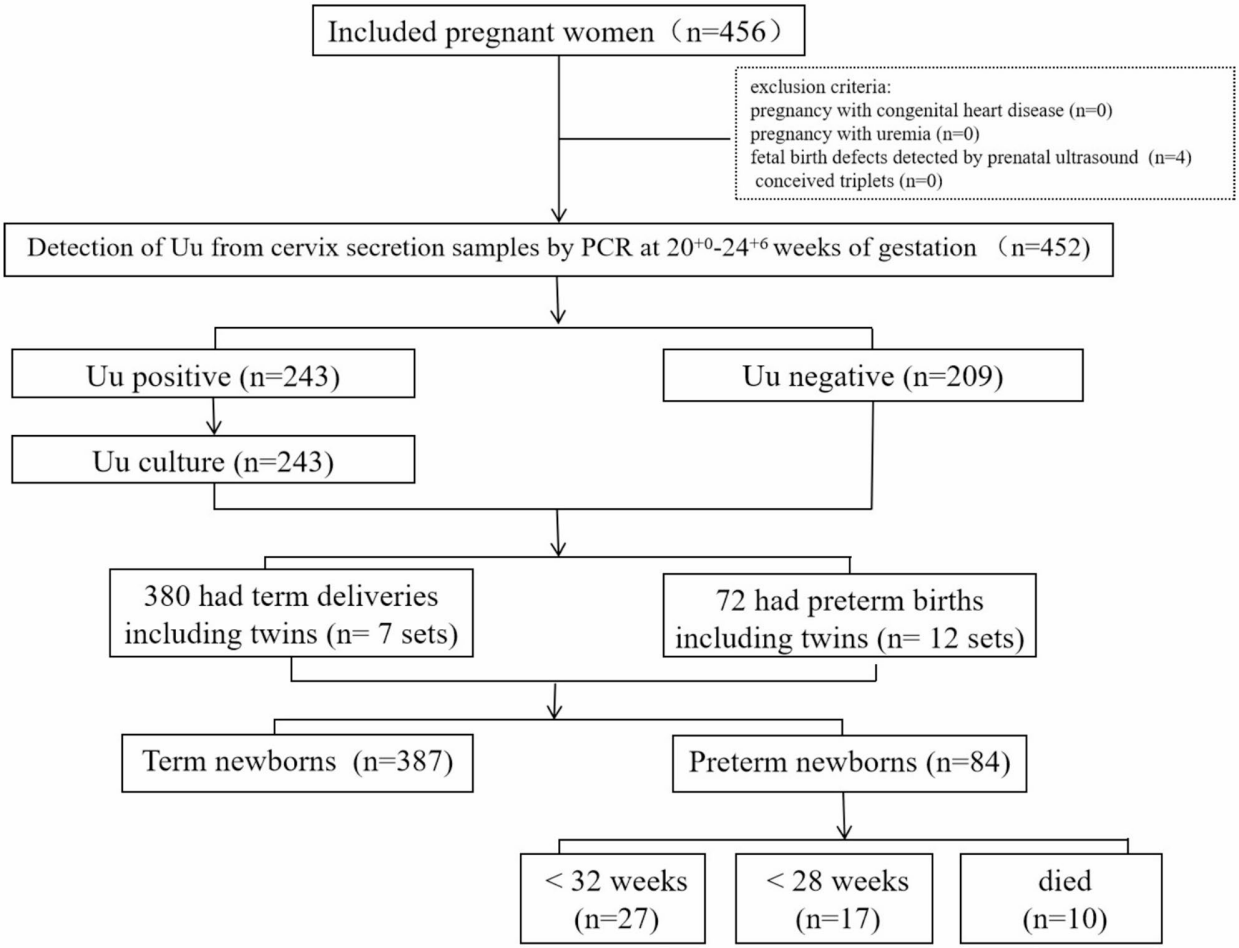
The flowchart of the study

A total of 456 pregnant women were enrolled in the study, with four cases excluded due to pregnancy terminations for congenital anomalies. The remaining 452 pregnant women who were included in the study completed the follow-up. The flowchart of the study is shown in Fig. 1. Among 452 pregnant women, 243 (53.7%) tested positive for Uu colonization in the cervical, as determined by PCR (Supplementary Table 1). The median Uu DNA load among Uu-positive pregnant women was 1.46 × 10^5^copies/mL. Comparison of demographic and obstetric characteristics between the Uu-positive and Uu-negative groups revealed no statistically significant differences in maternal age, body mass index (BMI), nulliparity, history of abortion, history of preterm birth, uterine malformation, cervical insufficiency, vaginal bleeding before 20 weeks of gestation, gestational diabetes mellitus, gestational hypertension, multifetal gestation, umbilical cord abnormalities, placental abruption, antenatal corticosteroid use, PROM, clinical chorioamnionitis, or maternal fever prior to delivery (all *P* > 0.05). However, the rate of conception via in vitro fertilization (IVF) was significantly lower in the Uu-positive group compared to the Uu-negative group (*P* < 0.05).

### Pregnancy and Neonatal Outcomes

Among the included participants, 380 (84.1%) had term deliveries, while 72 (15.9%) experienced preterm births, including 19 twin pregnancies. In total, 471 neonates were delivered, comprising 384 term infants (81.5%) and 84 preterm infants (17.8%). The earliest gestational age at delivery recorded was 23 weeks. Ten singleton preterm infants born between 23 and 25^+ 6^ weeks of gestation died following withdrawal of life-sustaining therapy. The proportion of preterm neonates undergoing discontinuation of life-sustaining treatment was significantly higher among mothers with cervical Uu colonization compared to those without colonization (3.7% vs. 0.5%, *P* = 0.045), indicating a potential association between maternal Uu positivity and adverse neonatal prognosis in extremely preterm births.

### Neonatal Characteristics and Early Complications

A comparison of neonatal outcomes based on maternal Uu colonization status is presented in Supplementary Table 2. No significant differences were observed in gestational age at birth, birth weight, 1- and 5-minute Apgar scores, or the incidence of common neonatal complications, including NRDS, neonatal sepsis, and VAP, between the Uu-positive and Uu-negative maternal groups (*P* > 0.05). These findings suggest that while maternal Uu colonization may be associated with an increased risk of preterm delivery and early neonatal death following life-sustaining treatment withdrawal, it does not appear to significantly affect the incidence of early neonatal complications among surviving preterm infants.

### Pregnancy and Neonatal Outcomes in Relation to High Uu load (≥ 10⁵ copies/mL) in cervical

In our cohort, the median Uu DNA load among Uu-positive pregnant women was 1.46 × 10^5^copies/mL. To further investigate the clinical significance of colonization burden, we stratified Uu-positive participants based on Uu load, using a threshold of ≥ 10⁵ copies/mL as indicated by our PCR results and supported by previous studies [8, 26]. Pregnancy outcomes were then compared between women with Uu load ≥ 10⁵ copies/mL and those who were either Uu-negative or had a load < 10⁵ copies/ml (Table 1).

**Table 1:** Comparison of the pregnancy outcomes of mothers with or without Uu load ≥ 10^5^ copies/mL in cervical

	Uu load ≥ 10^5^ copies/mL (*n* = 66)	Uu load < 10⁵ copies/mL or Uu-negative (*n* = 386)	χ^2^ or Z or T	*P*
WBC count before delivery (1*10^9)	10.20 ± 3.38	8.64 ± 2.62	3.381^a^	0.001**
Percentage of neutrophils before delivery (%)	72.50 (68.20, 78.90)	68.62 (73.45, 77.67)	-0.326^d^	0.745
CRP before delivery (mg/L)	1.74(0.05, 18.95)	3.50 (1.80, 5.80)	-1.215^d^	0.224
Term labor	51 (77.3%)	329 (85.2%)	2.667^b^	0.102
Preterm labor	15 (22.7%)	57 (14.8%)	2.667^b^	0.102
Gestational age (weeks)	38.86 (37.07, 39.86)	38.86 (37.43, 39.71)	-0.590^d^	0.555
Death occurring after discontinuation of life-sustaining therapy	5 (7.6%)	5 (1.3%)	10.276^b^	0.001**
< 32 weeks	11 (16.7%)	16 (4.1%)	15.734^b^	0.000**
< 28 weeks	9 (13.6%)	8 (2.1%)	20.823^b^	0.000**
PROM	24 (36.4%)	84 (21.8%)	6.609^b^	0.010*
Chorioamnionitis	9 (13.6%)	45 (10.8%)	0.371^b^	0.542

a.independent samples t-test ; b. Chi-square test. d. Mann-Whitney U test

*P < 0.05; **P < 0.01; *WBC *white blood cells, *CRP *C-reactive protein, *PROM *premature rupture of membranes

We found that the group with a Uu load ≥ 10⁵ copies/mL had higher maternal prenatal white blood cell (WBC) counts compared to the group with a Uu load < 10⁵ copies/mL or who were Uu-negative (10.20 ± 3.38 vs. 8.64 ± 2.62, *P* = 0.001). Participants with a Uu load ≥ 10⁵ copies/mL had a significantly higher incidence of spontaneous preterm birth at < 32 weeks (16.7% vs. 4.1%, *P* = 0.000) and < 28 weeks (13.6% vs. 2.1%, *P* = 0.000) than those in the combined low-load/Uu-negative group. Similarly, the rates of PROM (36.4% vs. 21.8%, *P* = 0.010) and of neonatal death following the discontinuation of life-sustaining therapy (7.6% vs. 1.3%, *P* = 0.001) were significantly elevated among women with a Uu load ≥ 10⁵ copies/mL. These findings suggest a dose-dependent association between Uu colonization burden and adverse pregnancy outcomes, particularly in the context of extreme prematurity.

Neonatal outcomes were also analyzed in relation to maternal Uu colonization burden (Table 2). Although the incidences of neonatal sepsis (2% vs. 0, *P* = 0.606), NRDS (9.7% vs. 7.4%, *P* = 0.542), and BPD (37.5% vs. 36.4, *P* = 1) were numerically higher in the Uu load ≥ 10⁵ copies/ml group compared to the Uu load < 10⁵ copies/ml and Uu-negative group, these differences did not reach statistical significance. No significant differences were observed in other neonatal outcomes, including gestational age at birth, birth weight, or Apgar scores, between the groups. These findings indicate that while a high cervical Uu load during mid-pregnancy is associated with an increased risk of preterm birth and PROM, its direct impact on major neonatal complications remains inconclusive in this cohort.

**Table 2:** Comparison of the newborn characteristics with or without Uu load ≥ 10^5^ copies/mL in cervical of mothers

Newborns (*n* = 471)				
	Uu load ≥ 10^5^ copies/mL of Mothers (*n* = 68)	Uu load < 10⁵ copies/mL or Uu-negative of Mothers (*n* = 403)	χ^2^ or Z or T	*P*
Male	35 (51.5%)	209 (51.9%)	0.004^b^	0.952
Gestational age (weeks)	38.86 (37.07, 39.86)	38.86 (37.43, 39.71)	-0.590^d^	0.555
Term newborns	51 (75.0%)	336(83.4%)	2.785^b^	0.095
Preterm newborns	17(25.0%)	67(16.6%)	2.785^b^	0.095
Birth weight (g)	3100 (2770, 3460)	3235 (2792, 3572)	-1.066^d^	0.286
< 2500 g of birth weight	14 (20.6%)	53 (13.2%)	2.637^b^	0.104
< 1500 g of birth weight	11 (16.2%)	16 (4.0%)	16.042^b^	0.000**
small for gestational age	3 (4.4%)	20 (5.0%)	-^e^	1.000
Apgar score at 1 min	9 (9,10)	9 (9,10)	-0.232^d^	0.816
Apgar score at 5 min	10 (10,10)	10 (10,10)	-1.636^d^	0.102
Need to be treat in NICU	17 (25.0%)	101 (25.1%)	0.000^b^	0.991
Sepsis	0	8 (2.0%)	-^e^	0.606
NRDS	5 (7.4%)	39 (9.7%)	0.371^b^	0.542
VAP	0	6(1.5%)	-^e^	0.600
< 32 weeks of gestational age	11(16.2%)	16(4.0%)	16.042^b^	0.000**
< 28 weeks of gestational age	9(13.2%)	8(2.0%)	21.167^b^	0.000**
BPD in premature < 32 weeks of gestational age	4(36.4%)	6 (37.5%)	-^e^	1.000

b. Chi-square test. d. Mann-Whitney U test. e. Fisher exact probability method

*P < 0.05, **P < 0.01

*NRDS *neonatal respiratory distress syndrome, *VAP *ventilator-associated pneumonia, *BPD *bronchopulmonary dysplasia

### The effects of different degrees of maternal Uu colonization on preterm infants born at < 32 weeks

We focused on infants born at < 32 weeks’ gestation due to their higher risk of complications and mortality. As shown in Table 3, the < 32 weeks group had significantly higher rates of cervical insufficiency, chorioamnionitis, placental abruption, PROM, and Uu load ≥ 10⁵ copies/mL than the ≥ 32 weeks group. Variables with *p* < 0.05 in the univariate analysis were included in the multivariate model. All variables had VIF values below 2, indicating no substantial multicollinearity. Subsequent multivariate logistic regression identified cervical insufficiency, chorioamnionitis, placental abruption, PROM, and Uu load ≥ 10⁵ copies/mL as independent risk factors for delivery at < 32 weeks (Table 4).

**Table 3:** Factors associated with premature delivery < 32 weeks

	< 32 weeks (*n* = 27)	≥ 32 weeks (*n* = 444)	T,χ^2^,or Z,	*P*
Mother’s age	31.41 ± 5.09	30.78 ± 4.47	0.704^a^	0.482
Number of pregnancies	1(1,3)	1(2,3)	-1.42^d^	0.156
Number of deliveries	0(0,1)	0(0,1)	-1.333^d^	0.182
Previous abortion	15 (55.6%)	186(41.7%)	1.924^b^	0.163
Previous premature delivery	2(7.4%)	12(2.7%)	-^e^	0.188
Uterine malformation	1(3.7%)	5(1.1%)	-^e^	0.300
Cervical insufficiency	8(29.6%)	16(3.6%)	35.650^b^	< 0.001**
In vitro fertilization	3(11.1%)	89(20.0%)	-^e^	0.324
Vaginal bleeding before 20 weeks of pregnancy	4(14.8%)	31(7.0%)	-^e^	0.131
Gestational diabetes mellitus	2(7.4%)	80(18.0%)	1.323^c^	0.250
Gestational hypertension	0	29(6.5%)	-^e^	0.197
Chorioamnionitis	7(25.9%)	44(9.9%)	6.762^b^	0.009**
abnormality of umbilical cord	4(14.8%)	95(21.4%)	0.327^c^	0.568
Placentae abruption	3(11.1%)	8(1.8%)	-^e^	0.020*
macrolide antibiotic used after 20 weeks of pregnancy	4(14.8%)	38(8.6%)	0.577^c^	0.447
PROM	16(14.5%)	11(3.0%)	20.627^b^	0.000**
Uu load ≥ 10^5^ copies/mL	11 (40.7%)	57 (12.8%)	16.042^b^	0.000**

a. independent samples t-test; b. Chi-square test. c. corrected Chi-square test; d. Mann-Whitney U test; e. Fisher exact probability method

*P < 0.05, **P < 0.01

*PROM *premature rupture of membranes

**Table 4:** Multivariate logistic regression analysis of risk factors for preterm infants < 32 week

	B	SE	Wald	*P*	OR	95% CI
Cervical insufficiency	2.062	0.601	11.722	0.001**	7.865	2.421 ~ 25.549
Chorioamnionitis	0.842	0.631	11.007	0.001*	4.740	1.883 ~ 11.932
Placentae abruption	2.572	0.873	8.678	0.003*	13.094	2.365 ~ 72.499
PROM	1.556	0.471	10.912	0.001**	4.740	1.883 ~ 11.932
Uu load ≥ 10^5^ copies/mL	1.732	0.522	11.007	0.001**	5.653	2.032 ~ 15.727

*P < 0.05, **P < 0.01. *SE *standard error, *OR *odds ratio, *95% CI *95% confidence interval, *PROM *premature rupture of membranes

### The effect of antibiotic treatment for maternal Uu load ≥ 10^5^ copies/mL on pregnancy outcomes

Among 243 pregnant women who underwent Ureaplasma culture, 127 (52.2%) were positive, among whom 12 (9.4%) showed resistance to one or more macrolides. Subsequent analysis focused on the high-load subgroup (Uu ≥ 10⁵ copies/mL, *n* = 66), of which 12 (18.1%) received treatment. As detailed in Table 5, the incidence of spontaneous preterm birth < 32 weeks, PROM, and chorioamnionitis did not differ significantly between treated and untreated women in this high-load group.

**Table 5:** The effect of antibiotic treatment for maternal Uu load ≥ 10^5^ copies/mL on pregnancy outcomes

	Treatment (*n* = 12)	Non-treatment (*n* = 54)	χ^2^	*P*
< 32weeks (*n* = 11)	3 (25.0%)	8 (14.8%)	-^e^	0.406
PROM (*n* = 24)	4 (33.3%)	20 (37.0%)	-^e^	1.000
Chorioamnionitis (*n* = 6)	0	6 (11.1%)	-^e^	0.582

e. Fisher exact probability method

## Discussion

Uu is considered a commensal of the adult urogenital tract [36]. In this study, 53.7% of the pregnant women were Uu positive in the genital tract. The impact of Uu colonization in the maternal genital tract on pregnancy outcomes remains controversial [5, 15]. Several studies have suggested that genital tract infection with Uu adversely affects the course of pregnancy to different degrees [5, 8]. In women with premature rupture of membranes, Uu is the most common pathogen found in amniotic fluid [37]. Miyoshi Y et al. [38] reported that maternal reproductive tract Uu infection was an independent predictor of preterm birth in patients with symptomatic threatened preterm birth labor and a short cervix. Abele-Horn et al. [7] reported that the degree of Uu colonization was correlated with adverse pregnancy outcomes. The critical insight from our study is the role of Uu load, with a median of 1.46 × 10⁵ copies/mL among positive women serving as a key differentiator.

The possible pathogenic mechanism of Uu involves the stimulation of phospholipase A2 activity in pregnant women, leading to the liberation of arachidonic acid. This promotes prostaglandin synthesis and increases the production of inflammatory factors such as IL-1, IL-6, and TNF-α, thereby aggravating the inflammatory response, affecting uterine contraction, and ultimately resulting in PROM [18]. In the case of PROM, vaginal colonization of Uu is associated with a considerable risk of ascending infection, especially at gestational ages below 28 weeks of gestation [39]. Uu can also exert its virulence through multiple banded antigen (MBA), IgA protease, and urease genes [40, 41]. By changing the size of the MBA domain, Uu can evade host immune recognition, weakening host defenses while still triggering a host antibody response [42]. Moreover, urease produced by Uu breaks down urea into ammonia, elevating the pH of vaginal secretions and promoting infection by other pathogens in the reproductive tract, thus contributing to the development of bacterial vaginosis [43]. In the context of the present study, we found that the group with a Uu load ≥ 10⁵ copies/mL had higher maternal prenatal WBC counts compared to the group with a Uu load < 10⁵ copies/mL or who were Uu-negative. The observed association between high Uu load (≥ 10⁵ copies/mL) and PROM, therefore consistent with these mechanistic pathways and reinforce the interpretation that high bacterial burden is a key driver of inflammation-related complications, whereas low-level colonization likely reflects a benign commensal state. We also observed more frequent development of chorioamnionitis in mothers in the Uu load ≥ 10⁵ copies/mL group than in the Uu load < 10⁵ copies/mL or Uu-negative group, although the difference was not significant. This finding warrants further investigation. However, a limitation of this mechanistic interpretation is that we did not comprehensively screen for other cervical pathogens. While Uu can alter the vaginal environment and promote co-infections [43], the specific contribution of Uu-driven inflammation independent of other pathogens requires further clarification. Regarding demographic and obstetric characteristics, no significant differences were observed between the Uu-positive and Uu-negative groups, except for a lower rate of conception via IVF in the Uu-positive group. This may be partly explained by the routine screening and treatment for cervical infections in women undergoing assisted reproduction prior to embryo transfer.

Preterm birth is a significant global problem, with infants born at lower gestational ages facing a greater risk of adverse medical and neurodevelopmental outcomes [2, 44]. This study primarily explores the relationship between maternal cervical colonization during the mid-trimester and adverse pregnancy outcomes, with a specific focus on the occurrence of preterm birth before 32 weeks of gestation. In this study, no statistically significant association was found between Uu-positive status in the maternal cervical and either preterm births before 37 weeks or those before 32 weeks. However, maternal Uu-positive in the cervical was associated with preterm infant death following withdrawal of life support at 23–25 weeks of gestation. Consistent with prior research [13], the results suggested an association between cervical Uu colonization and adverse pregnancy outcomes. Additionally, studies have demonstrated a correlation between the level of Uu colonization in the maternal cervical and adverse pregnancy outcomes [7, 21]. In our study, maternal cervical Uu load ≥ 10^5^ copies/mL was associated with spontaneous preterm delivery before 32 weeks of gestation, before 28 weeks of gestation, and PROM. Simultaneously, we observed the association between PROM and spontaneous preterm delivery before 32 weeks, which suggested that Uu load ≥ 10^5^ copies/ml may contribute to preterm birth through inflammation-induced PROM. These findings suggest that a positive Uu load ≥ 10^5^ copies/mL in the cervical may be associated with an increased risk of adverse pregnancy outcomes. These findings suggest that quantitative assessment of Uu colonization provides a more accurate reflection of infection-driven inflammation than mere presence or absence. High Uu load likely indicates active infection or inflammation, whereas low-level colonization may represent commensal status, which is less likely to impact adverse pregnancy outcomes. Therefore, the load of Uu in the maternal cervical is a parameter that should be considered in clinical management.It is important to note that our analysis was based on a relatively small number of early preterm birth events in the Uu load ≥ 10⁵ copies/mL group, which limits the statistical power and generalizability of the finding, and that larger, prospective studies are needed to confirm the role of cervical Uu load.

Studies have demonstrated that Uu positivity during pregnancy is associated with neonatal morbidities such as pneumonia, sepsis, NRDS, BPD, and central nervous system infection, especially in very preterm infants [45]. Uu-driven inflammation may be an underlying mechanism in the development of neonatal morbidities [46]. We observed more frequent development of sepsis, NRDS, and BPD in mothers in Uu load ≥ 10⁵ copies/mL group than in the Uu load < 10⁵ copies/mL or Uu-negative groups, although the difference did not reach statistical significance. This requires further confirmation in future research. These analyses were likely underpowered due to the limited number of endpoint events, and the fact that some critically ill newborns underwent withdrawal of life-sustaining therapy may have further influenced the incidence of these late-onset morbidities. This constitutes an important limitation in fully assessing the impact of Uu on neonatal complications.

Treatment strategies in Uu-colonized pregnant women have long been the subject of debate, weighing potential benefits against the risk of fostering antimicrobial resistance [47]. The reported resistance rate of genital Uu to macrolides ranges from 9.87% to 40.8% [48, 49]. In this study, 9.4% of the pregnant women were resistant to one or more macrolides. Kawakita T et al. [15] found the rate of treatment failure after the initial treatment of those who had a positive Uu culture was very high (78.6%), and there were no differences in adverse pregnancy outcomes, regardless of Uu culture results and whether pregnant individuals received treatment in a retrospective cohort study. Importantly, as Uu often behaves as a commensal organism, intervention based solely on its presence may not be clinically justified. Antimicrobial therapy is generally reserved for women with clinical symptoms of cervical infection or those with additional risk factors for preterm birth [32, 33]. In this study, we confirmed that a high Uu load (≥ 10⁵ copies/mL) is a significant risk factor for adverse perinatal outcomes. However, even within this high-risk subgroup, antibiotic treatment did not significantly reduce the incidence of spontaneous preterm birth < 32 weeks, PROM, or chorioamnionitis, indicating limited efficacy of empirical therapy. This lack of observed benefit may be explained by several factors, including the limited sample size of treated high-load cases, underlying antimicrobial resistance, delayed initiation of treatment, or inadequate antibiotic penetration into the intrauterine compartment. Therefore, this lack of observed benefit must be interpreted with caution, as a major limitation of our observational design was the lack of controlled treatment. Antibiotics were administered based on clinical discretion, not a standardized protocol, which introduces the potential for confounding by indication. Therefore, the true efficacy of targeted antibiotic therapy for high-load Uu colonization can only be definitively established through a randomized controlled trial.

The present study has several limitations. First, the sample size from four regional centers limits generalizability and precludes complete adjustment for all potential confounding factors. Second, the potential influence of co-existing genital tract pathogens on Uu colonization was not assessed; comprehensive pathogen screening is needed to accurately evaluate Uu’s specific impact on perinatal outcomes. Third, as cervical specimens for Uu testing were not collected at multiple time points during pregnancy, we could not determine the effect of persistent Uu colonization throughout gestation on perinatal outcomes. Fourth, the lack of control over antibiotic treatment for Uu colonization could potentially confound the results, as treatment decisions were based on routine clinical practice. Future studies should consider adjusting for antibiotic use or exploring its impact as a separate variable to better isolate the effects of Uu colonization on perinatal outcomes. Finally, the effects of Uu on complications in preterm infants and the effects of drug therapy on adverse pregnancy outcomes could not be thoroughly assessed, and larger prospective multicenter studies are required to validate these findings. Our further research will focus on these issues.

## Conclusion

Our study suggests that maternal cervical Uu colonization ≥ 10⁵ copies/mL may be associated with an increased risk of spontaneous preterm birth before 32 weeks. However, within the constraints of our observational design, antibiotic treatment did not demonstrate a clear benefit in reducing this risk. These findings underscore the necessity for future research, including large-scale prospective studies and randomized controlled trials, which integrate quantitative Uu assessment, host inflammatory markers, and optimized treatment timing to develop effective strategies for mitigating Uu-associated preterm birth.

## Supplementary Information

Supplementary Material 1.

## Data Availability

The all research data during this study were included in this article.

## Abbreviations

Uu: Ureaplasma urealyticum
PCR: Polymerase chain reaction
PROM: Premature rupture of membranes
NRDS: Neonatal respiratory distress syndrome
BPD: Bronchopulmonary dysplasia
NEC: Necrotizing enterocolitis
CRF: Scase report forms
VAP: Ventilator Associated Pneumonia
VIF: Variance inflation factor
OR: Odds ratio
CI: Confidence interval
BMI: Body mass index
IVF: In vitro fertilization
WBC: White blood cells
MBA: Multiple banded antigens
CRP: C-reactive protein
