# Supplementary material for: Association between maternal cervical Ureaplasma urealyticum colonization and adverse perinatal outcomes: a prospective multicenter cohort study
Source: BMC Pregnancy Childbirth. 2026 Feb 26;26:370. doi: 10.1186/s12884-026-08839-2 (PMC13041087; doi:10.1186/s12884-026-08839-2)
Supplement: Supplementary file 1 — Supplementary Material 1. [file 12884_2026_8839_MOESM1_ESM.doc]

Supplementary Table 1. Comparison of the maternal demographic and perinatal characteristics between women with and without Uu positive in cervical.

|  | Uu positive (n=243) | Uu negative (n=209) | χ^2^ or T or Z | *P* |
| --- | --- | --- | --- | --- |
| Maternal age | 30.48±4.71 | 31.18±4.29 | -1.652^a^ | 0.099 |
| Body mass index (BMI) | 25.99±3.52 | 26.11±3.68 | -0.350^a^ | 0.726 |
| Nulliparous | 116 (47.7%) | 104 (50.2) | 0.281^b^ | 0.596 |
| History of abortion | 100 (41.2%) | 93(44.9%) | 0.650^b^ | 0.420 |
| History of preterm birth | 8 (3.3%) | 4 (1.9%) | 0.819^c^ | 0.664 |
| Uterine malformation | 3 (1.2%) | 3 (1.4%) | 0.000^c^ | 1.000 |
| Cervical insufficiency | 10 (4.1%) | 12 (5.8%) | 0.664^b^ | 0.415 |
| In vitro fertilization | 34 (14%) | 44 (21.3%) | 0.4117^b^ | 0.042* |
| Vaginal bleeding before 20 weeks of pregnancy | 18 (7.4%) | 16 (7.7%) | 0.014^b^ | 0.907 |
| Gestational diabetes mellitus | 37 (15.3%) | 39 (18.8%) | 1.001^b^ | 0.317 |
| Gestational hypertension | 14 (5.8%) | 12 (5.8%) | 0.000^b^ | 0.996 |
| Multiple pregnancies | 9 (3.7%) | 10 (4.8%) | 0.340^b^ | 0.560 |
| abnormality of umbilical cord | 41 (17.5%) | 46 (22.3%) | 1.597^b^ | 0.206 |
| Placentae abruption | 4 (1.7%) | 6 (2.9%) | 0.795^b^ | 0.373 |
| Use of steroids | 33 (13.7%) | 33 (15.9%) | 0.448^b^ | 0.503 |
| PROM | 60(24.6%) | 38(18.1%) | 2.966^b^ | 0.085 |
| Chorioamnionitis | 30(12.3%) | 21(10.0%) | 0.593^b^ | 0.441 |
| Fever before delivery | 21 (8.7%) | 22 (10.6%) | 0.490^b^ | 0.484 |
| WBC count before delivery (1*10^9) | 9.00±2.76 | 8.72±2.84 | 0.913^a^ | 0.362 |
| Percentage of neutrophils before delivery (%) | 72.75 (67.82,78.00) | 74.00 (69.25,77.75) | -0.970^d^ | 0.260 |
| CRP before delivery (mg/L) | 3.95 (1.97, 6.20) | 3.45 (2.07,5.80) | 0.332^d^ | 0.795 |
| Term delivery (n=380) | 202 (83.1%) | 178 (85.2%) | 0.349^b^ | 0.555 |
| Preterm delivery (<37weeks) (n=72) | 41 (16.8%) | 31(14.8%) | 0.349^b^ | 0.555 |
| < 32weeks (n=27) | 18 (7.4%) | 9(4.3%) | 1.924^b^ | 0.165 |
| < 28weeks (n=18) | 13(5.3%) | 5(2.3%) | 2.570^b^ | 0.109 |
| Death occurring after discontinuation of life-sustaining therapy (n=10) | 9(3.7%) | 1(0.5%) | 4.015^c^ | 0.045* |

1. independent samples t-test ; b. Chi-square test. c. corrected Chi-square test; d. Mann-Whitney U test.

**P*<0.05.

PROM, premature rupture of membranes; WBC, white blood cells; CRP, C-reactive protein.

Supplementary Table 2. Comparison of the newborn characteristics with or without Uu positive in cervical of mothers

| Newborns (n=471) | | | | |
| --- | --- | --- | --- | --- |
|  | Uu positive in cervical of mothers (n=252) | Uu negative in cervical of mothers  (n=219) | χ^2^ or Z or T | *P* |
| Male | 136 (54.0%) | 108 (49.3%) | 1.016^b^ | 0.313 |
| Gestational age (weeks) | 38+6 (37+5, 39+6) | 38+6 (37+4, 39+6) | -0.435^d^ | 0.664 |
| Preterm newborns | 45 (17.9%) | 39 (17.8%) | 0.000^a^ | 0.989 |
| Birth weight (grams) | 2998.02±762.82 | 3069.00±655.34 | -1.075^a^ | 0.283 |
| Small for gestational age | 14 (5.6%) | 9 (4.1%) | 0.527^b^ | 0.468 |
| Apgar score at 1min | 10 (10,10） | 10 (10,10） | -0.398^d^ | 0.690 |
| Apgar score at 5min | 10 (10,10） | 10 (10,10） | -0.177^d^ | 0.860 |
| Need to be treated in NICU | 67 (26.6%) | 51 (23.3%) | 0.679^b^ | 0.410 |
| Sepsis | 3(1.2%) | 5(2.3%) | 0.756^c^ | 0.385 |
| NRDS | 29(11.5%) | 15(6.8%) | 3.003^b^ | 0.083 |
| Ventilator support | 11(4.4%) | 10(4.6%) | 0.011^b^ | 0.916 |
| VAP | 1(0.4%) | 5(2.3%) | 0.185^c^ | 0.159 |

a.independent samples t-test ; b. Chi-square test. c. corrected Chi-square test; d. Mann-Whitney U test.

**P*<0.05. NRDS, neonatal respiratory distress syndrome; VAP, ventilator-associated pneumonia
